# Retinal Pigment Epithelial and Outer Retinal Atrophy in Age-Related Macular Degeneration: Correlation with Macular Function

**DOI:** 10.3390/jcm9092973

**Published:** 2020-09-15

**Authors:** Maria C. Savastano, Benedetto Falsini, Grazia M. Cozzupoli, Alfonso Savastano, Gloria Gambini, Umberto De Vico, Angelo M. Minnella, Giorgio Placidi, Marco Piccardi, Stanislao Rizzo

**Affiliations:** 1 UOC Oculistica, Fondazione Policlinico Universitario A. Gemelli IRCCS, Largo A. Gemelli 8, 00168 Rome, Italy; mariacristina.savastano@gmail.com (M.C.S.); benedetto.falsini@unicatt.it (B.F.); alfonso.savastano@policlinicogemelli.it (A.S.); gambini.gloria@gmail.com (G.G.); umbertodevico@gmail.com (U.D.V); angelomaria.minnella@policlinicogemelli.it (A.M.M.); giorgioplacidi@libero.it (G.P.); piccmarc@tiscali.it (M.P.); stanislao.rizzo@unicatt.it (S.R.); 2 UOC Oculistica, Università Cattolica del Sacro Cuore, Largo A. Gemelli 8, 00168 Rome, Italy; 3 Consiglio Nazionale delle Ricerche, Istituto di Neuroscienze, Via G. Moruzzi 1, 56124 Pisa, Italy

**Keywords:** focal electroretinogram, sub-RPE illumination, age-related macular degeneration, outer retina and retinal pigment epithelium atrophy

## Abstract

The purpose of this study was to investigate the relationship between the retinal pigment epithelium (RPE) and outer retina changes, expressed in terms of sub-RPE illumination (SRI) on optical-coherence tomography (OCT), and central retinal function, measured by visual acuity and focal electroretinogram (fERG), in patients with non-exudative age-related macular degeneration (neAMD). In this retrospective study, 29 eyes of 29 patients affected by early (24.14%), intermediate (41.38%), and advanced (34.48%) neAMD were evaluated. All enrolled eyes were studied with OCT to measure the total area of SRI, by using an automated standardized algorithm. Visual acuity and fERG were assessed. The area of SRI was negatively correlated with fERG amplitude (r ≤ −0.4, *p* ≤ 0.02) and best-corrected visual acuity (BCVA) (r ≤ 0.4, *p* ≤ 0.04). Our results indicate that the severity of retinal pigment epithelium and outer retina atrophy (RORA), indirectly quantified through the detection of SRI areas by commercial OCT algorithms, is correlated with central retinal dysfunction, as determined by visual acuity and fERG, supporting the combined use of structural exams and functional tests as valid tools to detect the extent of RPE and photoreceptors’ disruption.

## 1. Introduction

Age-related macular degeneration (AMD) is one of the leading causes of central vision loss in older adults [1,2]. The real etiology of AMD is still not entirely understood, although some risk factors such as genetic susceptibility, age, diet, smoking, and environment are known to contribute to its occurrence [3].

According to the clinical classification by Ferris et al. [4], AMD can be categorized into three stages, namely, early, intermediate, and advanced. This classification is a categorical system based on the detection and extent definition of drusen and pigmentary abnormalities on color fundus photography (CFP). Ferris’ semiquantitative system has provided precise, standardized definitions for AMD lesions and it is still commonly used in clinical studies. However, since modern, high axial resolution optical-coherence tomography (OCT) has entered into common use, it has afforded the chance to detect and measure early lesions, even before they become clinically visible on CFP [5]. This is particularly true for geographic atrophy (GA) [6,7,8]. According to Guymer et al. [9], incomplete retinal pigment epithelium (RPE) and outer retina atrophy (iRORA) is defined on OCT as a region of signal hyper-transmission into the choroid, resulting from the interruption of the RPE. This finding corresponds to the zone of attenuation or disruption of the RPE, with evidence of the overlying photoreceptor degeneration, not exceeding 250 µm of width. The complete RPE deterioration leads to development of complete RORA (cRORA), that corresponds to the GA.

Hyper-transmission can be considered as an indirect sign of atrophy [5] and commercial OCT algorithms exist to quantify areas of increased choroidal signal due to RORA. However, some concerns have been raised with regard to considering choroidal hyper-transmission as a reliable surrogate of atrophy because it can sometimes be present even in the absence of photoreceptor degeneration or RPE loss [3]. For this reason, although OCT is paramount in the study of the macular atrophy, a multimodal imaging approach is always recommended in clinical practice to ensure that all the criteria of iRORA and cRORA are fulfilled [3,7].

Few studies have investigated the functional correlate of atrophic lesions at their different stages [10,11,12]. Therefore, it could be interesting to test whether, and to what extent, iRORA is compatible with relative preservation of visual acuity. Best-corrected visual acuity (BCVA) evaluation is deemed to be one of the easiest methods to assess the macular function but may not be sufficient to establish the real impairment of central retinal function. An objective method to assess the earliest degree of central visual dysfunction is focal electroretinogram (fERG) [13]. The fERG recorded from the central macular region of 18° in response to a 41 Hz flickering stimulus represents a fast, accurate, and non-invasive method to assess central retinal function in neAMD. Falsini et al. [14] reported that in AMD patients with soft drusen and/or early RPE defects, the fERG can detect a loss of retinal flicker sensitivity and response kinetics (response phase lag) in eyes with normal visual acuity. In addition, the abnormalities of the fERG response tend to be more profound and severe as the AMD lesions become more severe [15].

The goal of this study was to evaluate the correlation between a major parameter provided by automated OCT algorithm analysis, that is the area of sub-RPE illumination (SRI) within the macular region, and the degree of macular dysfunction, assessed by visual acuity and fERG.

## 2. Methods

This study was approved by the Ethics Committee/Institutional Review Board of the Catholic University (A/10992008). This research adhered to the tenets of the Declaration of Helsinki and informed consent was obtained from all patients, after full and detailed explanation of the goals and procedures of the study. All the clinical, imaging, and electrophysiological data reported in this study were retrospectively re-evaluated, in light of Ferris’ classification of RORA.

### 2.1. Subjects

We retrospectively reviewed the medical records of consecutive patients who had visited the retina service of the Department of Ophthalmology at Policlinico Universitario A. Gemelli IRCCS (Istituto di Ricovero e Cura a Carattere Scientifico)—Università Cattolica del Sacro Cuore (Roma, Italy), between May 2014 and December 2016.

Inclusion criteria were: (1) patients who had undergone OCT, OCT angiography (OCTA), and fERG, and (2) diagnosed with early, intermediate, or advanced neAMD (the diagnostic criteria for these disease stages are described in the following section).

Patients with the following conditions were excluded from the study: (1) metabolic systemic disorders (uncontrolled systemic hypertension, diabetes, and kidney failure), (2) refractive errors exceeding 4 spherical and 1 cylindrical diopters, (3) significant optical media opacities, (4) concomitant retinal or optic nerve disorders, (5) history or signs of exudative AMD (eAMD), (6) inability to collaborate during imaging and electrophysiological testing, and (7) OCT scans with a signal strength < 7.

### 2.2. Multimodal Imaging and Visual Function Test Methods

All patients underwent a complete ophthalmologic examination, including measurement of best-corrected visual acuity, determination of intraocular pressure, fundus stereoscopic evaluation with slit-lamp biomicroscopy, and non-contact 78 D lens, color fundus photography (CFP), optical coherence tomography (OCT), and OCT angiography (OCTA).

BCVA was assessed by the Early Treatment Diabetic Retinopathy Study (ETDRS) charts and reported in corresponding Snellen units.

Color fundus photographs of the posterior pole were obtained using a Cobra non-mydriatic fundus camera (Cobra HD Fundus Camera, CSO, Florence, Italy) after medical dilation of the pupil (tropicamide 1.0%). High-definition 5 Line Raster and 6 × 6 mm Macular Cube 512 × 128 scan protocols were performed using OCT (Zeiss Cirrus 5000-HD-OCT Angioplex, sw version 10.0, Carl Zeiss, Meditec, Inc., Dublin, CA, USA).

**Figure 1:**
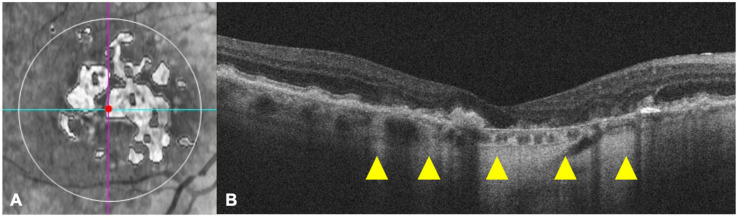
The sub-retinal pigment epithelium (RPE) platform (**A**), automatically provided by the Advanced RPE analysis software, shows the sub-RPE illumination (SRI) regions within the circular area of 5 mm (white outline) around the fovea (centered by the red point at the intersection between the cyan horizontal and purple vertical slice navigators). The horizontal optical-coherence tomography (OCT) scan passing through the fovea (corresponding to the cyan line on enface image) clearly evidences some zones of light hyper-transmission within the choroid, as indicated by the yellow arrowheads (**B**).

Advanced RPE analysis was performed on Macular Cube 512 × 128 scan data from all the patients. The Advanced RPE analysis software was used to automatically determine areas of RPE elevation (mm^2^), consistent with drusen and RPE detachments, and areas of sub-RPE illumination (mm^2^) for increased light penetration through atrophic RPE and choriocapillaris, by the means of a RPE elevation map and sub-RPE slab tools, respectively. The RPE elevation map detects the separation between the RPE segmentation line following the curvature of the eye and a line fitting to the curvature deformations of the RPE, averaged over a circular area of either 3 or 5 mm around the fovea. The SRI identifies bright areas of increased light transmission beneath the RPE, indicating RPE atrophy, averaged over a circular area of 5 mm around the fovea by the automated OCT software. (Figure 1).

The foveal center on the OCT fundus image was identified by using an automated fovea localization algorithm. When the algorithm failed for significant tissue disruption, two separate examiners (M.C.S. and G.M.C.) identified the fovea manually. In case of disagreement, a third author (B.F.) was consulted to reach consensus. Similarly, the images provided by the Advanced RPE analysis and Macular Thickness analysis software were analyzed by two independent retina expert investigators (M.C.S. and G.M.C.) to check the reliability of retinal layer segmentation. If segmentation errors were observed, manual correction was performed and accomplished by two independent examiners, and interobserver percentage of agreement was 86% (95% confidence interval = 74–98%). In cases of disagreement, both readers re-analyzed the images, and a consensus was obtained. Three types of mistakes were identified: inner limiting membrane (ILM) delineation error, RPE delineation error, and sub-RPE illumination segmentation error on enface image. In order to exclude eyes with eAMD, OCTA was performed, and patients with macular neovascularization (MNV) [16] or suspected MNV were discarded.

**Figure 2:**
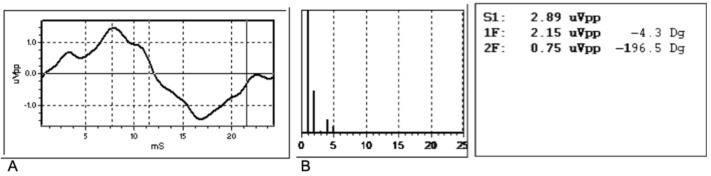
Example of raw (**A**) and analyzed (**B**) results of a typical focal electroretinogram (fERG) response obtained from a normal subject. The Fourier analysis (a discrete Fourier series represented as a histogram) is shown below the signal. In the Fourier histogram, every number indicates the even and odd multiples of the fundamental harmonic at 41 Hz. uVpp: peak-to-peak amplitude in µV; ms: milliseconds; S1. Total signal amplitude; 1F: first harmonic (41 Hz; first bar); 2F: second harmonic (82 Hz; second bar); Dg: phase value in degrees.

Cone-mediated focal macular ERG testing was performed according to a previously published technique [14,17]. Briefly, fERG was recorded from the central 18° region using a uniform red field stimulus superimposed on an equiluminant steady adapting background, used to minimize stray-light modulation. The stimulus was generated by a circular array of eight red light emitting diodes (LEDs) (k maximum 660 nm, mean luminance 93 cd/m^2^) presented on the rear of a Ganzfeld bowl (white-adapting background, luminance: 40 cd/m^2^). A diffusing filter in front of the LED array made it appear as a circle of uniform red light. fERGs were recorded in response to the sinusoidal 95% luminance modulation of the central red field. Flickering frequency was 41 Hz. Patients fixated monocularly at a 0.25° central fixation mark, under the constant monitoring of an external observer. Subjects underwent a preadaptation period of 20 min to the stimulus mean illuminance. fERGs were recorded by an Ag-AgCl electrode taped on the skin over the lower eyelid. A similar electrode, placed over the eyelid of the contralateral patched eye, was used as a reference (interocular recording). fERG signals were amplified (100,000-fold), bandpass filtered between 1 and 100 Hz (6 decibels/octave (dB/ oct)), and averaged (12-bit resolution, 2 kHz sampling rate, 200–600 repetitions in 2–6 blocks). Off-line discrete Fourier analysis quantified the peak-to-peak amplitude and phase lag of the response fundamental harmonic (first harmonic) at 41 Hz. Figure 2 shows an example of raw and analyzed results of a typical fERG response obtained from a normal subject. The Fourier analysis (a discrete Fourier series represented as a histogram) is shown below the signal. In the Fourier histogram, every number indicates the even and odd multiples of the fundamental harmonic at 41 Hz. It can be seen that most of the signal energy is concentrated at the fundamental frequency (1F, 41 Hz), with a minor contribution at the second harmonic (2F, 82 Hz) and at higher harmonics. The 1F component is considered representative of the response [14]. Noise level was estimated by collecting the same responses when the stimulus was occluded. The amplitude of the noise 1F in this patient was 0.10 microVolts (µV) (peak-to-peak) compared to a response 1F amplitude of 2.15 µV. The mean noise 1F amplitude in our patients was 0.07 µV (standard deviation, SD: 0.02).

### 2.3. Definition of neAMD Lesions

According to the AMD clinical classification by Ferris et al. [4], the presence and dimensions of drusen as well as pigment abnormalities (hyper- or hypo-pigmentation) are the criteria for establishing the stage of AMD. The early and intermediate stages of AMD are also considered as non-exudative AMD (neAMD), while the late stage is defined by the presence of eAMD and/or geographic atrophy (GA).

Early neAMD is characterized by medium (<63 and ≤125 µm) drusen and no pigmentary abnormalities, while intermediate neAMD is identified by the presence of large (>125 µm) soft drusen, observable as deposits between retinal pigment epithelium (RPE) and Bruch’s membrane and/or pigmentary abnormalities. Sometimes, lipofuscin-like retinal deposits can be localized above the RPE, and are thus called pseudodrusen [18].

With regard to GA, in the context of advanced AMD, we integrated Ferris’ classification with Guymer’s definition of iRORA and cRORA, based on OCT-specific findings.

iRORA is characterized by: (1) a region of signal hyper-transmission into the choroid not exceeding 250 µm, (2) a corresponding zone of attenuation or disruption of the RPE, with or without persistence of basal laminar deposits (BLamD), and (3) evidence of overlying photoreceptor degeneration, i.e., subsidence of the inner nuclear layer (INL) and outer plexiform (OPL), presence of a hypo-reflective wedge in the Henle fiber layer (HFL), thinning of the outer nuclear layer (ONL), disruption of the external limiting membrane (ELM), or disintegrity of the ellipsoid zone (EZ). When the RPE and outer retina atrophy involves more than 250 µm, then it is called cRORA, which corresponds to GA.

### 2.4. Statistical Analysis

The values obtained by the morpho-functional assessment of the patients were blinded to the clinicians so that the statistical analysis and interpretation of the data were unbiased. Data are presented as mean and standard deviation (SD), unless otherwise specified. Sample size of the study was determined by taking into account the published variability of OCT according to Guymer et al. [8] and fERG parameters in AMD. Assuming a standard deviation of 25% for either OCT and fERG parameters, a sample size of 29 patients provided an 80% power at an alpha of 0.05 to detect a correlation coefficient of at least 0.4 between OCT and functional measurements. Statistical analysis was performed by using Pearson’s correlation coefficient and linear regression analysis, assuming a normal bivariate distribution. A *p* < 0.05 was considered statistically significant.

## 3. Results

A group of 29 patients (41.38% men and 58.62% women; mean age ± SD, 68.55 ± 9.29 years; age range, 54–87; 29 eyes) affected by early (E, 24.14%), intermediate (I, 41.38%), and advanced (A, 34.48%) neAMD, out of an original cohort of 70 patients assessed in this study, was retrospectively evaluated. Forty-one patients were excluded, inasmuch they did not meet the inclusion criteria. The mean age of the included patients was 69 (±8.8) years. There were 17 females and 12 males. The mean BCVA was 0.6 (SD: 0.2). Demographic and clinical characteristics of the study cohort are reported in Table 1.

**Figure 3:**
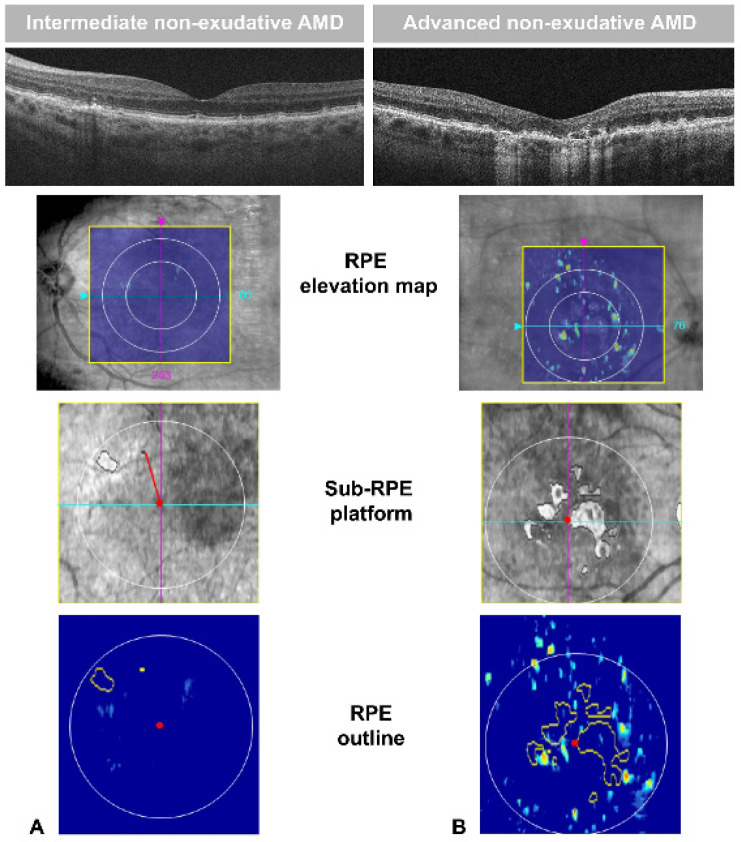
Images of B-scan, retinal pigment epithelium (RPE) elevation map, sub-RPE platform, and RPE outline of two patients with intermediate (**A**) and advanced (**B**) non-exudative age-related macular degeneration (neAMD). Drusen are associated with variable degree of RPE elevation (colored dots appearing within the 6 × 6-mm yellow square of RPE elevation map) as well as changes in sub-RPE platform (areas of increased sub-RPE illumination (SRI) within the 5-mm circle outlined in white). In the SRI platform, the red line represents the SRI Fovea Distance, connecting the fovea center (red point) to the nearest SRI area. The software provided an automated measure of RPE elevation, averaged over the area within a circle of either 3 and 5 mm around the fovea. Similarly, the SRI area (the sum of areas outlined in yellow in the RPE outline) was automatically calculated by the algorithm.

**Figure 4:**
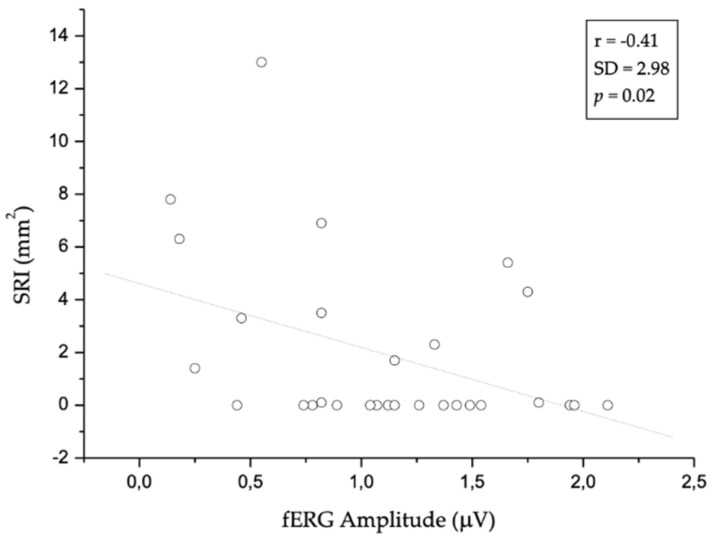
The scatter plot shows the statistically significant (*p* ≤ 0.02) correlation between focal electroretinogram (fERG) and sub-retinal pigment epithelium illumination (SRI). In particular, SRI is negatively correlated with fERG: the increase in SRI area is related to reduced fERG amplitude. Dotted line indicates linear regression. SD: Standard Deviation.

**Figure 5:**
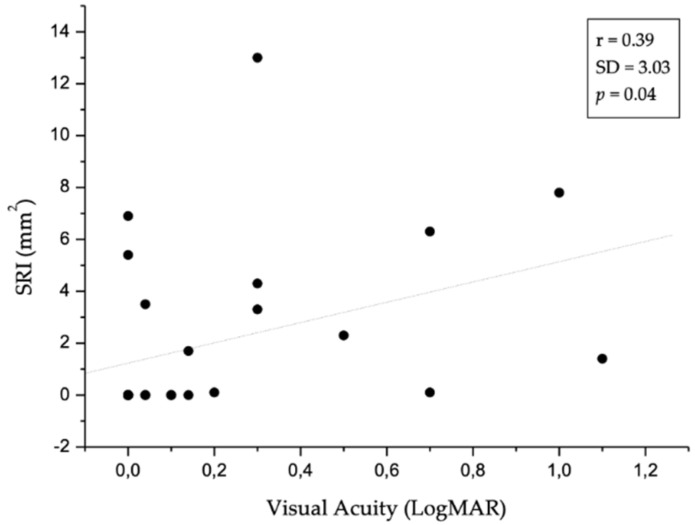
The scatter graph demonstrates the statistically significant (*p* ≤ 0.04) inverse correlation between visual acuity (Logarithm of minimum angle of resolution, LogMAR) and sub-retinal pigment epithelium illumination (SRI). The visual acuity decreases with increasing SRI area. Dotted line indicates linear regression. SD: Standard Deviation.

**Figure 6:**
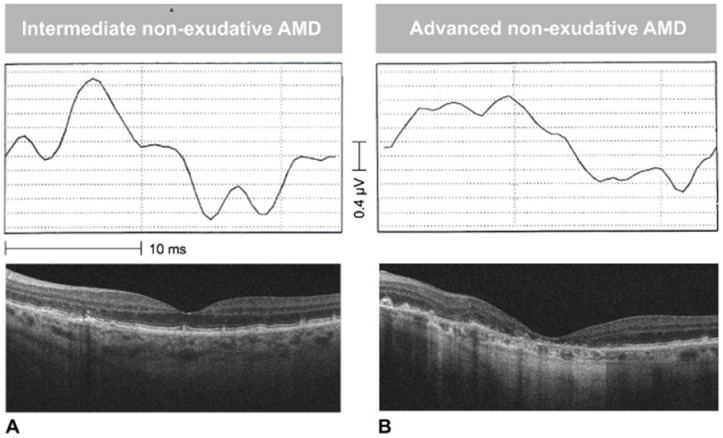
Examples of focal electroretinogram (fERG) responses in intermediate (**A**) and advanced (**B**) non-exudative age-related macular degeneration (neAMD). The difference between the two responses is evident by looking at the fERG signal, which appears wider in the left panel than in the right one.

Figure 3 shows the image of an RPE elevation, sub-RPE platform, and RPE outline in an intermediate and advanced neAMD stage. Differences in drusen size and RORA extension are visually appreciable in the RPE elevation map and sub-RPE platform, respectively. The OCT software also provided an automated measure of RPE elevation and SRI areas. In Figure 4 and Figure 5, the values of SRI area are reported as a function of fERG amplitude and visual acuity of patients, respectively. In our neAMD patients, SRI was negatively correlated with both BCVA (LogMAR) and fERG functional parameters, with an r ≤ 0.4 and −0.4, at a *p* ≤ 0.04 and *p* ≤ 0.02, respectively. Figure 6 shows the fERG difference response in intermediate and advanced neAMD.

No correlation was found between the RPE elevation area and BCVA and fERG amplitude, respectively (*p* = not significant, ns).

## 4. Discussion

In our study, we used the automatic OCT major parameter, sub-RPE illumination (SRI), as a quantitative index, expressed in mm^2^, of either iRORA or cRORA, with respect to the 250 µm width cut-off [5] used to discriminate between the former and the latter. As mentioned in the Methods Section, the measurement obtained by SRI analysis corresponds to the total area of bright pixels underlying the regions of RPE impairment, hence it is dependent upon the light hyper-transmission through the choroid, due to the atrophy of RPE and outer retina [5].

OCT offers new diagnostic tools thanks to the evolution of numerous standardized automated algorithms providing qualitative and quantitative analyses, that may be more sensitive than conventional methods [8]. This represents a crucial point, as OCT technology allows to disclose atrophic changes at an early stage, when a complete loss of RPE and outer retina has not yet occurred, and to monitor their gradual enlargement. As a consequence, a new rigorous classification of macular atrophy has been proposed [5,9], based on microscopic OCT findings.

Previous studies have reported other OCT parameters as possible biomarkers for visual acuity reduction, such as foveal enface ellipsoid zone (EZ) interruption. In particular, Kiernan et al. [19] demonstrated a significant correlation between EZ disconnection and visual acuity reduction, highlighting the importance of B-scan and enface OCT study in everyday clinical practice. To explore new biomarkers of neAMD progression, Pappuru et al. [20] evaluated the relationship between outer retinal thickness substructures and visual acuity in eyes with neAMD. They found that the integrity of the EZ and the external limiting membrane (ELM) was the most significant finding for predicting visual acuity impairment. However, the correlation was only moderate and did not fully explain the variability in visual acuity. Lujan et al. [21] were among the first to highlight OCT accuracy in identifying and quantifying the areas of GA. Further, they observed that the size and shape of these areas correlated to the areas of GA seen on fundus autofluorescence images.

We attempted to determine retrospectively, in a blinded fashion, whether SRI could be a good index of macular atrophy, and as such, associated with changes in central retinal function in neAMD patients. Our results indicate that the extent of iRORA and cRORA, assessed by the means of the quantitative OCT parameter SRI, is correlated with central retinal dysfunction, as determined by fERG and visual acuity. Based on the present findings, we suggest that a morpho-functional assessment of neAMD eyes may provide more sensitive and specific results, and it may be useful for patients’ follow-up, clinical studies, and trials testing new drugs with a personalized therapeutic target.

A limitation of this study is the relatively small sample size and the lack of follow-up. Nonetheless, the results unequivocally showed an inverse association between the OCT morphometric parameter SRI, and central retinal function. These findings support the use of combined analysis of SRI and fERG/visual acuity parameters to monitor the progressive RPE and outer retina disruption, relate it to macular function deterioration, and estimate the rate of progression from iRORA to cRORA, thus paving the way to personalized medicine in the follow-up and treatment of neAMD patients. Future perspectives will envisage to analyze SRI and fERG parameters in a longitudinal study.

## Figures and Tables

**Table 1:** Demographic and Clinical Features of Studied Patients.

	Age (Years)	Sex	Disease Stage	fERG Amplitude (µV)	BCVA (LogMAR)	RPE-Elevation 3 mm Circle Area (mm^2^)	RPE-Elevation 5 mm Circle Area (mm^2^)	SRI Area (mm^2^)	SRI Fovea Distance (mm)
**1**	83	M	E	1.12	0	0	0	0	XXX
**2**	54	F	I	1.94	0	0	0	0	XXX
**3**	73	M	A	0.82	0	1.4	2.8	6.9	0.1
**4**	60	F	E	1.96	0.04	0	0	0	XXX
**5**	64	F	E	1.49	0	0	0	0	XXX
**6**	77	F	I	0.89	0.1	0.5	0.5	0	XXX
**7**	63	F	E	1.54	0	0	0	0	XXX
**8**	70	M	I	0.82	0.2	0.1	0.3	0.1	0.9
**9**	59	M	A	1.66	0	1.2	2.8	5.4	0.5
**10**	63	F	I	1.37	0	0	0	0	XXX
**11**	70	F	A	1.33	0.5	0.7	1.8	2.3	0
**12**	63	M	A	0.14	1	0.2	1.2	7.8	0
**13**	74	F	A	1.75	0.3	2.1	3.2	4.3	0
**14**	79	M	A	0.82	0.04	2.2	4.1	3.5	0
**15**	54	F	E	1.15	0.1	0	0	0	XXX
**16**	55	M	E	1.43	0	0	0	0	XXX
**17**	68	F	I	2.11	0	0.3	0.3	0	XXX
**18**	72	M	I	1.80	0.7	0	0	0.1	0.1
**19**	69	M	I	0.78	0	0	0.1	0	XXX
**20**	81	F	A	1.15	0.14	0.2	0.2	1.7	0
**21**	74	F	A	0.55	0.3	0.2	0.3	13	0
**22**	56	M	I	0.46	0.3	0	0	3.3	0
**23**	64	F	I	1.26	0	0	0	0	XXX
**24**	87	M	A	0.74	0.14	6.3	10.9	0	XXX
**25**	59	F	E	1.07	0	0	0	0	XXX
**26**	64	F	I	0.44	0	0	0	0	XXX
**27**	78	F	I	1.04	0.04	0	0	0	XXX
**28**	80	F	A	0.18	0.7	0.1	0.7	6.3	0
**29**	75	M	I	0.25	1.1	0	0.4	1.4	1.5

RPE-elevation 3 and 5 mm Circle Area: automated measure of RPE elevation area, averaged over a circular area of both 3 and 5 mm around the fovea. Sub-RPE Illumination (SRI): bright areas of increased light transmission beneath the RPE, indicating RPE atrophy. Fovea Distance: distance of SRI areas to the fovea center. When the SRI area is 0, the measurement of distance to the fovea is not applicable and the output given by the automated software is XXX. A: advanced; BCVA: best-corrected visual acuity; E: early; F: female; fERG: focal electroretinogram; I: intermediate; LogMAR: Logarithm of minimum angle of resolution; M: male; RPE: retinal pigment epithelium; SRI: sub-RPE illumination; µV: microVolts.
